# Spinal osteophyte as a possible source of left atrial tachycardia

**DOI:** 10.1016/j.hrcr.2023.04.009

**Published:** 2023-04-24

**Authors:** Patrik Tóth, Bálint Szilveszter, Péter Perge, Csaba Fejér, László Gellér, Klaudia Vivien Nagy

**Affiliations:** Heart and Vascular Center, Semmelweis University, Budapest, Hungary

**Keywords:** Atrial fibrillation, Atrial tachycardia, Ablation, Pulmonary vein isolation, Osteophyte, Posterior wall, Pulsed-field ablation

**Key Teaching Points**

• Extracardiac structures may cause different arrhythmias like atrial fibrillation or atrial tachycardia.
• Preprocedural imaging like computed tomography angiography is a useful tool to reliably visualize cardiac and extracardiac anatomy.
• In challenging situations, a correct assessment of the risk-benefit ratio is of paramount importance.

## Introduction

Pulmonary vein isolation (PVI) is the most efficient and widely accepted method for treating atrial fibrillation (AF). Both the European Society of Cardiology^1^ and American College of Cardiology^2^ guidelines consider PVI as a standard and effective initial treatment option. Generally, preprocedural planning includes some form of imaging modality to exclude left atrial (LA) appendage thrombus. Cardiac computed tomography (CT) angiography reliably depicts LA anatomy, which can be used as a guide during the ablation procedure. Moreover, coronary arteries and other adjacent structures are also visible on CT images, eventually highlighting possible anatomical difficulties. The proximity of vertebra and vertebral osteophytes may cause such challenging situations. Other anatomical structures, like tumors,^3^ cysts,^4^ hernias,^5^^,^^6^ or aneurysms^7^ may compress the LA. Prior case reports evaluating the role of extracardiac structures described the presence of osteophytes causing compression on the LA without any substantial clinical relevance.^8^^,^^9^ In 1 case a possible arrhythmogenic effect of a spinal osteophyte causing AF has been hypothesized.^10^ However, focal arrhythmias with a similar suspected pathogenesis have not been described before.

## Case report

The 73-year-old male patient with arterial hypertension first presented in our clinic in August 2019 with symptomatic paroxysmal AF (EHRA class IIa). No previous fever, infectious disease, or thyroid disease was detected. Echocardiography revealed reduced left ventricular ejection fraction of 40%, diffuse hypokinesis, dilated left and right atrium (50 × 65 mm and 66 × 76 mm, respectively), dilated right ventricle (54 mm), and elevated pulmonary artery systolic pressure (29 + 10 mm Hg). Transthoracic echocardiography did not reveal any signs of mechanical deformation or extrinsic compression. Catheter ablation was scheduled for September 2019 after obtaining informed consent from the patient.

Preprocedural CT angiography showed conventional LA anatomy and nonsignificant coronary artery stenosis of the left main and left anterior descending artery. Furthermore, a solid pulmonary nodule in the S5 segment of the left lobe, signs of chronic obstructive pulmonary disease, and degenerative abnormalities of the lower thoracal vertebrae were also reported. Successful PVI was performed using a 3D anatomical mapping system (CARTO 3®; Biosense Webster, Irvine, CA) and irrigated ablation catheter (SmartTouch; Biosense Webster, Irvine, CA) with a point-by-point radiofrequency ablation technique. No early or late complications were observed.

The patient remained free from any atrial arrhythmia until October 2022, when he presented with rapid AF with aberrant conduction (right bundle branch block and left anterior hemiblock). He had no complaints of back pain or recent trauma. After the failure of pharmacological cardioversion using oral amiodarone, the patient was scheduled for a repeat procedure using pulsed-field ablation technology.

**Figure 1:**
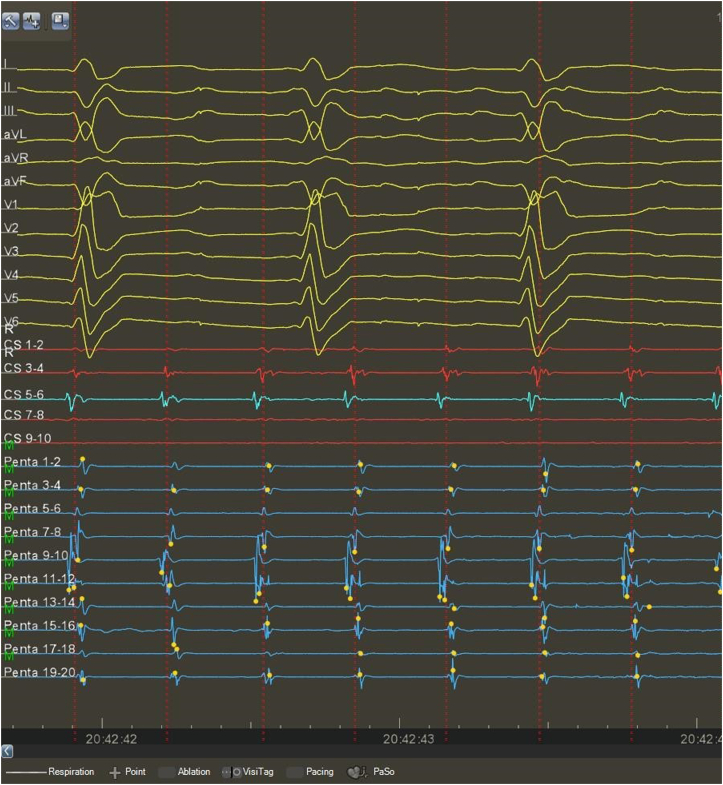
Twelve-lead surface electrocardiogram and intracardiac electrogram signals of the clinical arrhythmia at the site of earliest activation during pulmonary vein isolation using PentaRay catheter and CARTO electroanatomical mapping system (Biosense Webster, Irvine, CA).

The clinical arrhythmia was recurrent AF. During the procedure, the first observed rhythm was atrial tachycardia (AT) with a cycle length of 300 ms and 78 beats per minute ventricular frequency (Figure 1). The transseptal puncture was performed with intracardiac echocardiographic, fluoroscopic, and pressure monitor guidance. Owing to the stout interatrial septum, administration of SL0 or Agilis (Abbott Laboratories, Chicago, IL) sheaths could not be achieved, despite several guidewire replacements. Therefore, we performed a septostomy with an 8 × 40 mm Passeo X35 balloon (Biotronik, Berlin, Germany), successfully accessing the LA. Intravenous heparin was administered based on the patient’s weight, with a target activated clotting time (ACT) of 350 seconds or above. After a total of 25,000 international units of heparin, the ACT was 287 seconds. Although the ACT was below the optimal target time, we opted not to administer more heparin before the mapping to avoid serious bleeding complications. An additional ACT measurement was scheduled just before the insertion of the FaraDrive (Boston Scientific, Marlborough, MA) sheath.

**Figure 2:**
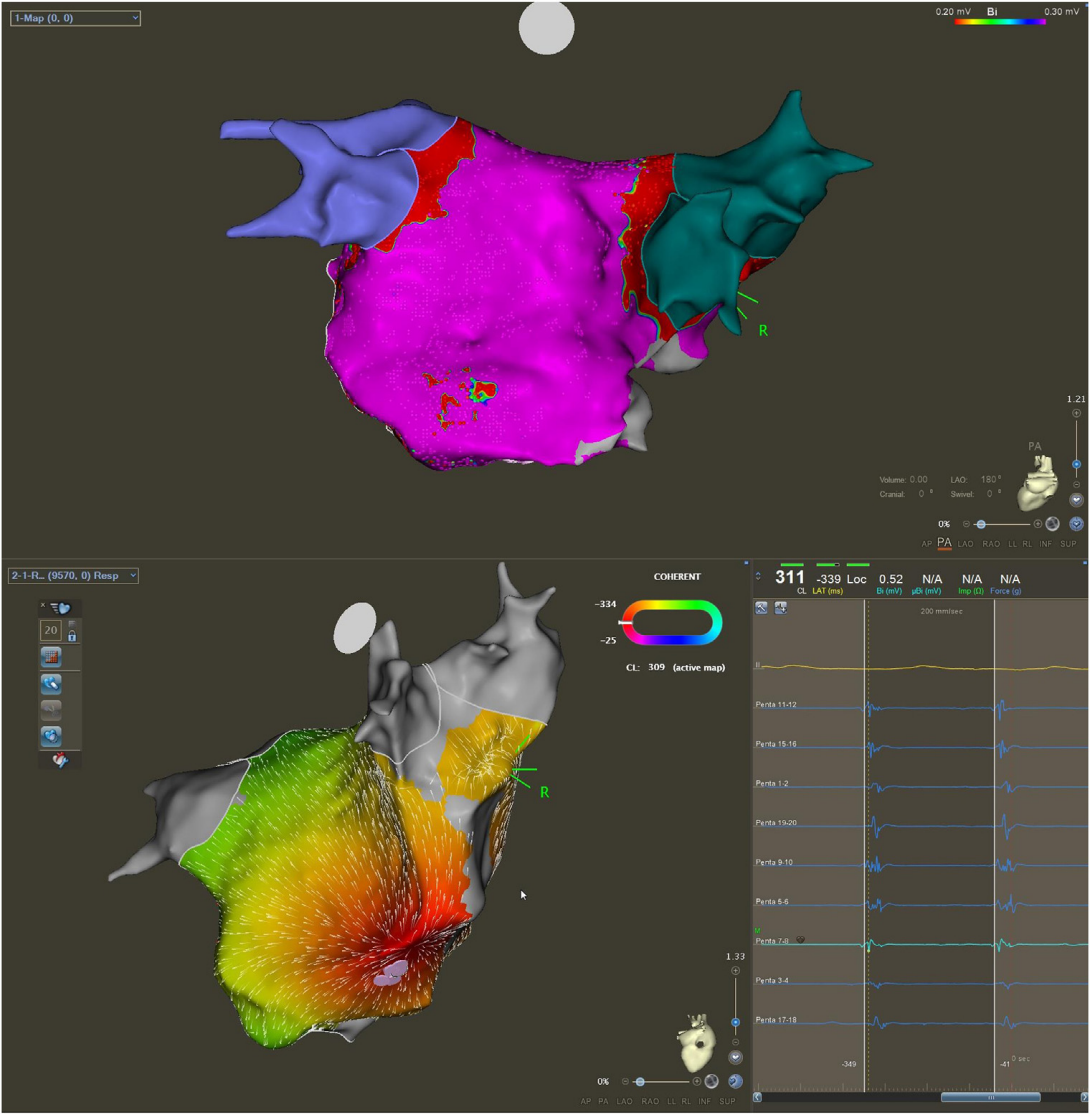
Voltage map (**A**) and activation map (**B**) of repeat pulmonary vein isolation using CARTO system (Biosense Webster, Irvine, CA). Activation map (**B**) showing earliest activation on the posterior wall of the left atrium from the tip of the compression caused by the vertebral osteophyte. Intracardiac electrograms of earliest activation signals are indicated in the lower right corner.

There was no sign of any extrinsic compression on the intracardiac echocardiography. An electroanatomical map, voltage map, and activation map were created using a PentaRay (Biosense Webster, Irvine, CA) catheter (Figure 2). All pulmonary veins were isolated. The earliest, fragmented activation was observed on the posterior wall of the LA on a large protrusion (Figure 2).

**Figure 3:**
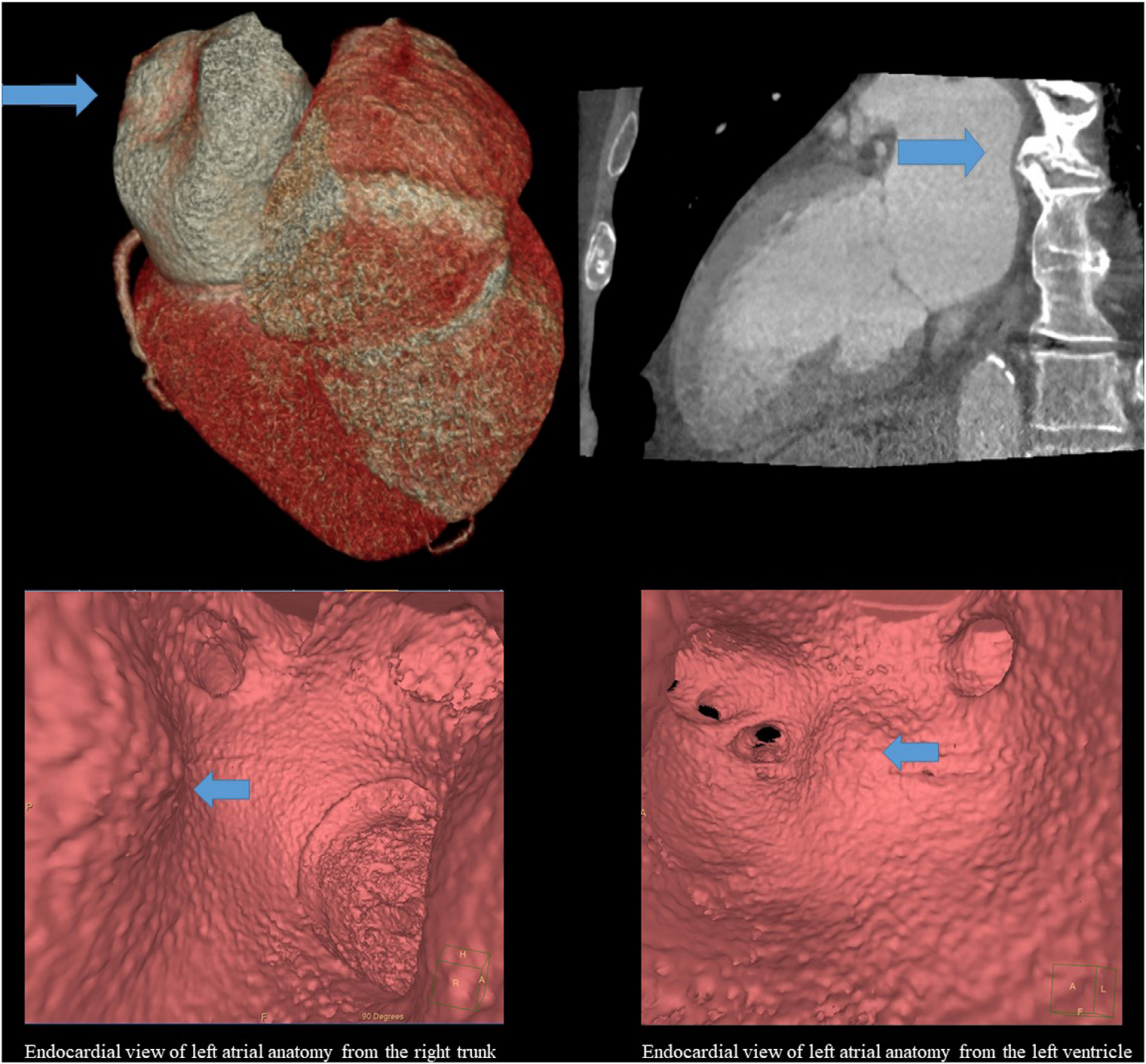
Cardiac computed tomography angiography showing compression on the posterior wall of left atrium caused by vertebral osteophyte (**A:** 3D volumetric rendering of the heart, posterior view and 2D view of the left atrium and ventricle). The blue arrows indicate the site of compression on the posterior wall of left atrium caused by vertebral osteophyte as depicted on the endocardial views from the right common trunk and from the left ventricle (**B**).

After careful reevaluation of CT images and a radiologist consult, we identified a prominent, heavily calcified, claw-shaped anterior thoracic osteophyte at T8–T9 at the right lateral contour of the vertebrae along with signs of disc degeneration, compressing the posterior wall of the LA (Figure 3). Based on the electroanatomical map, activation map, and earliest local activation, we assumed that the origin of the AT is at the tip of this protrusion caused by the spinal osteophyte. The average LA wall thickness on CT, measured at the roof, was 3.3 mm, compared to the posterior wall, which was 1.5–1.7 mm.^11^ Owing to the high risk of wall injury and lack of experience with the FaraPulse system, we did not perform any ablation.

Finally, electrical cardioversion was performed owing to the persistent nature of the arrhythmia. Radiofrequency ablation was not attempted owing to the possibly thinned atrial wall on the CT images. Moreover, pivoting to another source of energy would have taken a long time, increasing the risk of complications in a patient with a high dose of anticoagulant.

After a successful electrical cardioversion, the patient is asymptomatic. After careful consideration we decided to wait with any ablation until arrhythmia recurrence and confirmation of atrial wall dimensions. No complications occurred during or after the procedure. During the latest follow-up visit the patient had no AF or AT recurrences. He had no complaints of chest pain, syncope, dyspnea, or palpitations. A 24-hour Holter ECG monitoring and echocardiography were scheduled. If the patient experiences persistent episodes of AT or AF, we plan to perform a radiofrequency catheter ablation with preprocedural magnetic resonance imaging of the LA and adjacent structures.

## Discussion

Osteophytes causing atrial protrusion has been described before.^8^, ^9^, ^10^ Osteophytes are bony growths that develop owing to excessive bone and fibrocartilage cell production and are typically found in the degenerated cervical or thoracic spine at locations experiencing prolonged stress. Rare intracardiac structures like a right atrial appendage aneurysm^12^ or a patent foramen ovale^13^ may be sources of AT. The focal arrhythmic effect of ganglionated plexi as a result of vagal stimulation might also be a rare cause of AT.^14^ However, to the best of our knowledge, an osteophyte as a source of focal AT has not been described before.

During the initial PVI, the patient underwent the same preprocedural imaging protocol where degenerative abnormalities were only described at the lower thoracal region. There was no clear-cut evidence of any vertebral osteophyte. After an unremarkable procedure, the patient remained asymptomatic and in sinus rhythm for 3 years. After a documented AF recurrence, a pulsed-field ablation was scheduled. While during the initial procedure the transseptal puncture was easy to perform, the second time a septostomy was necessary to successfully access the LA. Another interesting finding was the short ACT, despite high doses of heparin, which was also a novel observation compared to the first ablation.

During the redo procedure, we observed a regular arrhythmia with left atrial origin (Figure 1). We performed activation mapping to visualize the activation sequence in the LA, which confirmed focal activation on the posterior wall. Based on the underlying pathomechanism, we identified focal AT as the clinical arrhythmia (Figure 2). The map clearly identified the focus of the AT, which was also confirmed by the earliest local activation on the posterior wall of the LA; therefore, we did not perform any additional pacing maneuvers (including entrainment).

The lack of experience with the FaraPulse system had a major impact on our decision to stop the procedure after mapping. Only 1 similar case was described before, where Morales and colleagues^8^ used radiofrequency energy. Moreover, the potential thinning of the posterior wall on the tip of the protrusion could not be excluded, which further elevated the procedural risks, including rupture of the atrial wall. Although pulsed-field ablation technology has remarkable tissue selectivity,^15^ there are no data regarding such proximity of a bone structure. We concluded the overall risk outweighed the patient benefit and stopped the procedure.

## Conclusion

In rare cases, the proximal extracardiac structures may lead to myocardial wall distortion, causing different arrhythmias. Although 3D anatomical mapping systems provide details about the target location of arrhythmias, they do not visualize the extracardiac structures. Therefore, preprocedural imaging might be helpful to reliably assess the adjacent anatomy.
